# Assessing Liver Fibrosis Using the FIB4 Index in the Community Setting

**DOI:** 10.3390/diagnostics11122236

**Published:** 2021-11-29

**Authors:** Albert Blanco-Grau, Pablo Gabriel-Medina, Francisco Rodriguez-Algarra, Yolanda Villena, Rosa Lopez-Martínez, Salvador Augustín, Mònica Pons, Luz-Maria Cruz, Ariadna Rando-Segura, Belen Enfedaque, Mar Riveiro, Ernesto Casis, Roser Ferrer-Costa, Maria Buti, Francisco Rodriguez-Frias

**Affiliations:** 1 Clinical Biochemistry (Clinical Laboratories), Vall d’Hebron University Hospital, 08035 Barcelona, Spain; alblanco@vhebron.net (A.B.-G.); pgabriel@vhebron.net (P.G.-M.); yvillena@vhebron.net (Y.V.); rosam.lopez@vhebron.net (R.L.-M.); lmcruz@vhebron.net (L.-M.C.); ecasis@vhebron.net (E.C.); 2 Clinical Biochemistry Research Group, Vall d’Hebron Institut de Recerca (VHIR), 08035 Barcelona, Spain; 3 Blizard Institute, Barts and The London School of Medicine and Dentistry, London E1 2AT, UK; f.rodriguez-algarra@qmul.ac.uk; 4 Liver Unit, Department of Internal Medicine, Vall d’Hebron University Hospital, 08035 Barcelona, Spain; salva.augustin@gmail.com (S.A.); monica.pons@vhir.org (M.P.); mmriveiro@vhebron.net (M.R.); mbuti@vhebron.net (M.B.); 5 Clinical Microbiology (Clinical Laboratories), Vall d’Hebron University Hospital, 08035 Barcelona, Spain; a.rando@vhebron.net; 6 Community and Primary Care Management, Catalan Institute of Health, 08028 Barcelona, Spain; menfedaque.bcn.ics@gencat.cat; 7 Centro de Investigación Biomédica en Red de Enfermedades Hepáticas y Digestivas (CIBEREHD), 28029 Madrid, Spain; 8 Medicine and Science School, Universitat Autònoma de Barcelona, Bellaterra, 08193 Barcelona, Spain; 9 Department of Internal Medicine, Vall d’Hebron University Hospital, 08035 Barcelona, Spain

**Keywords:** chronic liver disease, liver fibrosis, metabolic associated fatty liver disease (MAFLD), screening, fibrosis 4 score

## Abstract

Liver disease is frequently asymptomatic, challenging early identification in the primary care setting. The fibrosis 4 (FIB4) index is a liver fibrosis biomarker that is a potential alternative to liver biopsy for diagnosing and managing liver disease. This study aimed to calculate the FIB4 index for screening individuals at high risk of liver disease at the community level. This was a retrospective real-world study analyzing blood and serum test results from a central laboratory. The primary outcome was the number of individuals within each risk category for hepatic fibrosis: high risk (FIB4 ≥ 3.25) and low risk (FIB4 < 1.3). The analysis included samples from 31,753 patients, of which 18,102 were aged 40 to 75 years. In these patients, the FIB4 index had been explicitly requested in 1852 (10.2%) cases and estimated ad hoc in the rest. Of the 263 (1.5%) cases with FIB4 ≥ 3.25, the FIB4 index was requested in 46 (17.5%), and 52 (19.8%) showed evidence of liver fibrosis in their medical records, while the rest did not report any data regarding liver fibrosis. FIB4 is a simple score that can play a role as a “red flag” for early identification of patients at high risk of advanced liver fibrosis and their referral to specialized care.

## 1. Introduction

Chronic liver disease is a major cause of mortality globally and leads to a substantial healthcare burden. The causes of liver disease may vary depending on the region and patient’s age, but viral hepatitis infections, metabolic-associated fatty liver disease (MAFLD), and alcohol consumption are the most common etiologic agents. Regardless of the cause, chronic liver disease often presents asymptomatically until advanced phases, when liver damage is irreversible and therapy can only slow or stop progression of the disease [1,2].

Early diagnosis of liver disease, particularly in the primary care setting, is a mainstay to change this undesirable scenario. Traditionally, liver disease is suspected from the elevation of hepatic enzymes and further confirmed by liver biopsy. However, frequently, patients with advanced liver disease do not show liver enzyme alterations precluding suspicions for deciding its diagnosis.

MAFLD is the leading cause of chronic liver disease worldwide, affecting 17% to 46% of adults in high-income countries. The prevalence of MAFLD strongly correlates with the global burden of obesity and type 2 diabetes mellitus (T2DM), the most common risk factors for this condition [3]. In normal weight individuals, the prevalence of MAFLD may reach 7%, including those with normal levels of liver enzymes [3,4]. While MAFLD has a relatively benign prognosis, patients with non-alcoholic steatohepatitis (NASH) are at risk of developing progressive fibrosis and eventually cirrhosis. These patients often remain asymptomatic until they develop end-stage liver disease. Liver fibrosis is the strongest predictor of clinically meaningful outcomes, including decompensation, liver cancer, and overall mortality [5].

Liver biopsy is an invasive procedure that leads to complications [6] and has a significant diagnostic error rate [7,8], limiting its use for screening liver disease in the general population. In recent years, non-invasive markers or techniques have been proposed for the study of liver disease, such as positron emission tomography (PET), magnetic resonance (MR) imaging, and, especially, transient elastography (TE). TE assesses liver stiffness [9] and has proven to be cost-effective for a population screening of liver fibrosis [10], but it is not available in all healthcare settings, and some factors may alter its performance (e.g., obesity, post-prandial testing).

Alternatively, the degree of liver fibrosis can be measured using well-established panels of serum markers determined in routine assessments. Current international guidelines for hepatitis C treatment support the use of biochemical indexes to assess the extent of hepatic fibrosis [9,11,12,13]. Additionally, some authors have suggested their utility for identifying individuals at high risk of severe liver disease [14]. Indexes based on serological markers perform well in the identification of advanced fibrosis/cirrhosis. Although their utility in resolving intermediate degrees of fibrosis is limited [9], for some (e.g., aspartate aminotransferase-to-platelet ratio index [APRI] and fibrosis 4 [FIB4] score), several cut-off values have been proposed to establish different stages of fibrosis in patients with chronic hepatitis C or NASH [15,16,17,18,19,20,21]. The FIB4 index, originally proposed to help assess hepatic fibrosis in patients with human immunodeficiency virus (HIV) and hepatitis C virus (HCV) coinfection [22], can be calculated from age and three parameters obtained in routine laboratory assessments: alanine aminotransferase (ALT), aspartate aminotransferase (AST), and platelet count.

Taken together, current evidence highlights the importance of identifying high-risk patients in routine practice [23]. Given its simplicity, availability, and affordability, the FIB4 index has been proposed by the World Health Organization and various scientific associations as an alternative method to identify liver fibrosis in countries with limited access to specific fibrosis serum panels or electrographic physical methods [24]. However, areas with central laboratories that process many blood samples in a routine primary care setting have the opportunity to use FIB4 as a sentinel index to screen individuals at high risk of liver disease at the community level. Therefore, we aimed to calculate the FIB4 index in adult subjects whose blood was drawn under routine care.

## 2. Materials and Methods

### 2.1. Study Design and Data Sources

In this retrospective real-world study, we analyzed blood and serum samples from non-selected patients from the city of Barcelona covered by the public healthcare system and regularly monitored at the analytical level at the Vall d’Hebron Hospital Clinical Laboratories between 15 April and 27 June 2019. These laboratories provide in vitro diagnostic services to Vall d’Hebron University Hospital (1200 beds) and to the entire population of Barcelona managed by the public healthcare system (1.3 million people attending approximately 100 healthcare centers). All samples with the parameters available (i.e., ALT, AST, platelet count, and age) for FIB4 estimation were included for the analysis. If the same patient showed several results, all samples except the first one, based on the date it was ordered, were excluded. Additionally, all samples with ALT ≥ 400 IU/L were excluded to prevent high FIB4 levels due to an artifact caused by acute liver disease [25,26].

To simplify and increase the efficiency of requesting analytical tests in a routine care setting, the Catalonian Health System has implemented a set of Primary Care Protocols (PCPs) with multiple tests. Community clinicians can request those PCPs that better suit each clinical situation or diagnostic suspicion. PCPs 18 and 19 include the FIB4 index. For this study, the FIB4 index was estimated in all patients with ALT, AST, platelet count, and age available, regardless of whether FIB4 was explicitly requested (Table S1, Supplementary Materials).

All samples were collected during routine care, and data were handled according to the General Data Protection Regulation 2016/679 on data protection and privacy for all individuals within the European Union. This study was approved by the Ethics Committee of Vall d’Hebron Hospital.

### 2.2. Study Outcomes and FIB4 Estimate

The primary outcome was the number of patients with an FIB4 index within each risk stratum: high risk (FIB4 ≥ 3.25) and low risk (FIB4 < 1.3). For individuals with high-risk values, we also analyzed the percentage of samples in which the FIB4 index had been requested.

However, in order to focus the study on those patients most likely to develop hepatic fibrosis, and because older age may lead to falsely high FIB4 results, a subanalysis was conducted in samples from patients aged 40 to 75 years (i.e., the age range recommended by the American College of Cardiology/American Heart Association for managing blood cholesterol concerning the risk factors for atherosclerotic cardiovascular disease, which shares common characteristics with MAFLD and NASH) [27].

The FIB4 index was estimated as follows (age in years, ALT and AST in IU/L, and platelet count in 10^9^/L):

$$FIB4\;index=\frac{\left(age\;\times \;AST\right)}{platelet\;count\;\times \;\sqrt{ALT}}$$

### 2.3. Statistical Analysis

Quantitative variables were described using measures of central tendency (mean and median) and skewness (standard deviation [SD] and range), whereas categorical variables were described as the frequency and percentage over available data. Means of laboratory parameters from patients in each stratum of the FIB4 index were compared using the Student’s t-test. The threshold for statistical significance was set at a two-sided alpha value of 0.05. “R” package version 3.6.1 was used for database debugging and selecting the cases to be included in the analysis and for all statistical analyses.

## 3. Results

### 3.1. Patients Included and Their Distribution According to the FIB4 Index

During the study period, our laboratory received blood and serum samples from 31,753 patients (41.8% males) whose FIB4 was calculated. Of them, 18,102 (43.2% males) were aged between 40 and 75 years. The FIB4 estimate was requested by the physician in 1852 (10.2%) cases, while in the remaining 16,250, the index was calculated ad hoc for the purpose of the study. An FIB4 ≥ 3.25 was detected in 293 (1.6%) cases (66.9% males); in 50 (2.7%), FIB4 was originally requested. Twenty-six of the ad hoc cases had platelet aggregates (an artifact situation for FIB4 calculation), and four with muscle disturbances were reassigned to the low-risk groups once the platelet count was measured in citrated blood. After these corrections, the number of patients with advanced fibrosis across risk strata was 263 (1.5%) with FIB4 ≥ 3.25, 11,091 (61.2%) with FIB4 < 1.3, and 6748 (37.2%) with 1.3 ≤ FIB4 < 3.25.

Table 1 summarizes the distribution of the main biochemical parameters in the entire sample, in the subpopulation aged 40 to 75 years, and across all FIB4 cut-off values. AST and ALT levels significantly increased with higher FIB4 values. The corresponding comparisons for glucose and triglycerides were limited by the remarkable number of unavailable data.

Table S1 shows the distribution of FIB4 cut-offs for each PCP. Of all requested PCPs, 43,513 (94.2%) corresponded to six well-defined clinical settings: diabetes, liver diseases, hypercholesterolemia, arterial hypertension, thyroid pathology, and anemia, with an additional group named “Basic Health studies”, which included several health conditions. In these clinical settings, 519 cases of FIB4 ≥ 3.25 were detected when FIB4 was analyzed ad hoc, due to the “screening” strategy (Table 2).

More than one PCP was frequently requested in the same patient, resulting in 1871 PCP combinations in 15,396 patients. In 756 (4.9%) cases, only the Basic Health study (PCP24) was requested, precluding estimation of the FIB4 index (Supplementary Materials Table S2).

### 3.2. Medical Records of Patients with FIB4 ≥ 3.25

Table 3 summarizes the frequency of FIB4 requests and evidence of liver fibrosis in patients’ medical records (i.e., confirmed by TE, MR, or sonography) among individuals with FIB4 ≥ 3.25. Medical records (MedRecs) were available for 242 patients, of which 190 (79%) lacked evidence of liver fibrosis, suggesting that it was not previously assessed. Only 13 (28%) cases with requested FIB4 and 39 (18%) with ad hoc estimated FIB4 reported evidence of liver fibrosis.

Seventy-two (38%) cases without reported evidence of liver fibrosis (57 without a FIB4 request) showed conditions strongly associated with a high likelihood of liver fibrosis, including alcohol consumption, HCV chronic infection, HBV chronic infection, and MAFLD (Table 3). The FIB4 index could be an indicator for emphasizing lifestyle changes, improving the management of other diseases, or decreasing alcohol consumption in the primary care setting.

Of 52 patients with evidence of fibrosis in their MedRecs, 10 had been diagnosed with T2DM, arterial hypertension, dyslipidemia, and/or obesity. Of the 190 patients with no MedRec evidence of fibrosis, 67 had the same diagnosis, but FIB4 was requested only for 10 of them (Table 3). Altogether, in 77 cases with several well-known and diagnosed risk factors, FIB4 may be a useful tool for understanding liver function.

Overall, 185 (81%) cases showed PCP combinations related with suspected metabolic syndrome. However, metabolic syndrome was reported in the MedRec for only 92 of them. In fact, in 13 cases in which no PCPs related with metabolic syndrome were requested, the presence of related diagnoses was identified, including eight cases with arterial hypertension (four with diabetes) and five with other comorbidities. In fact, after reviewing their MedRecs, 111 (42%) of the 263 patients with FIB4 ≥ 3.25 had records associated with metabolic syndrome.

## 4. Discussion

In highly populated areas, major laboratories often centralize thousands of routine care tests. In this regard, our laboratory provides in vitro diagnostic services to the entire population of the city of Barcelona managed by public healthcare (1.3 million people). This scenario facilitates the implementation of screening programs in community health, which faces significant challenges due to the wide range of clinical situations.

Most patients with asymptomatic liver disease are managed in the primary care setting, a condition that often remains undetected. In light of this scenario, the systematic use of blood tests as a screening tool may become a mainstay to uncover hidden cases. The utility of blood tests for identifying advanced fibrosis was recently confirmed by Chan et al. in a retrospective cohort of 759 patients with biopsy-proven MAFLD, even in a two-step strategy by combining blood tests with Liver Stiffness Measurements [28]. After this validation, and taking advantage of our activity as a central laboratory for the whole Barcelona city population, we thought it would be interesting to directly apply the said blood test, such as the FIB4 index, as a screening process for the risk of advanced liver fibrosis in a real-world cohort.

In this study, we selected the FIB4 ≥ 3.25 cut-off to indicate possible advanced fibrosis [22,29]. This cut-off, originally validated for HCV infection, has also been validated for other pathologies, like hepatitis B [30] and MAFLD [31].

Our strategy allowed us to identify 263 (1.5%) cases of potentially advanced fibrosis that should be evaluated for liver disease [31]. The significant increase in aminotransferase levels observed when FIB4 ≥ 3.25 seems to agree with other findings indicating that this index evidences liver pathology [32,33]. Moreover, FIB4 < 1.3 was observed in 61.4% of individuals who could be directly managed in a primary care setting [31]. Thus, focusing on the range of 40–75 years of our population, it can be inferred that this screening strategy, in one year, could allow identification of nearly 1500 patients at high risk of advanced fibrosis [31].

Given the asymptomatic nature of the liver disease, the rate of FIB4 requests observed was very low, irrespective of FIB4 values. Most cases with a high risk of advanced fibrosis were detected without prior FIB4 request and after requesting PCPs corresponding to specific clinical situations (i.e., diabetes, liver pathology, hypercholesterolemia, arterial hypertension, thyroid pathology, and anemia), suggesting the involvement of metabolic syndrome. In fact, after reviewing patients’ medical records, we found that 42% of cases with FIB4 ≥ 3.25 were associated with metabolic syndrome.

In our study, only a minority of patients with FIB4 ≥ 3.25 had previously documented evidence of liver fibrosis, suggesting a lack of suspicion about this pathology. According to MedRec, in 190 (72%) of these cases, FIB4 was the first sign of possible progression to liver fibrosis and, of these, only 72 (38%) had conditions associated with a higher risk of liver fibrosis (alcohol consumption, viral hepatitis, or liver steatosis). The lack of records highlights the difficulties of community health doctors in obtaining clinical information. Our data suggest that most of these patients could have a major problem due to advanced liver fibrosis not previously identified. Therefore, in 62% of these cases, FIB4 ≥ 3.25 represented the first warning to consider the presence of liver fibrosis, mainly as the result of the inclusion of this index (83%). A similar benefit was observed in viral hepatitis C and B cases, especially in those without previous evidence of liver fibrosis. Thus, referring this group of patients to more specialized care must be considered. We can assume that in non-requested FIB4 ≥ 3.25 cases detected in PCP studies related with liver pathology, this index would probably be requested at some point during liver disease monitoring. However, this value was likely to remain unnoticed in the remaining cases, preventing specialist referral.

MAFLD is considered the liver manifestation of metabolic syndrome [34] and might be strongly associated with FIB4 ≥ 3.25 cases [35]. Based on MedRec, of the 263 cases with FIB4 ≥ 3.25, only 16 had a prior MAFLD diagnosis, 13 without documented evidence of liver fibrosis. This finding seems to reinforce the usefulness of the FIB4 index as a first warning to explore the possibility of liver fibrosis. Three of the main six clinical scenarios observed in PCP requests (diabetes, dyslipidemia, and hypothyroidism) have been associated with MAFLD [3]. These data strongly support the benefit of this inexpensive screening strategy, allowing patients with these clinical conditions to receive optimal management.

Nevertheless, in patients aged 40–75 years, intermediate FIB4 values (≥1.3 to 3.25) would probably have been detected in about 33,000 yearly cases, which could be reclassified using the enhanced liver fibrosis (ELF) serum marker. However, the ELF test is currently expensive, and the cost-effectiveness of this measure is yet to be confirmed (27). In this regard, some partial strategies targeting high-risk groups of patients, such as those with metabolic syndrome, could be explored. For instance, a more intensive follow-up can be recommended by measuring FIB4 periodically to detect a high risk of advanced liver fibrosis before clinical onset [14] and keeping common associated risk factors under control, such as T2DM, obesity, and dyslipidemia.

Routine screening for liver fibrosis is controversial and, from a practical point of view, this study’s design represents a kind of “community screening”. The EASL–EASD–EASO Guidelines [4] have raised concerns regarding the need for community MAFLD screening and highlighted the need for validated cost-utility studies on extensive screening programs because of their prognostic implications of MAFLD progression to NASH―particularly associated with advanced fibrosis―indicating that it should be identified in patients at risk. Additionally, they [3] have established that MAFLD and NASH should be suspected in patients with T2DM, indicating that the clinical decision must be supported by FIB4 or MAFLD fibrosis scores, in addition to vibration-controlled transient elastography (VCTE), which is more sensitive than FIB4 but much more complex and expensive [36,37]. A combination of both strategies could be applied: initial FIB4 screening at the community level, followed by an additional testing, such as VCTE, in specialized facilities for FIB4 ≥ 3.25 cases, or even for FIB4 ≥ 1.3 cases. Other recent non-invasive, multiparametric, ultrasound-based tools are able to quantify both steatosis and fibrosis [38,39,40]. This strategy can be complemented by ELF testing [31].

The systematic implementation of the strategy described in this study could help to identify liver fibrosis in the general population, which accounts for 1.5% according to our study, representing a sound argument for engaging policymakers in addressing the serious problem of liver fibrosis related with different liver pathologies (viral hepatitis, MAFLD, etc.). Given its real-world nature, our study lacks additional demographic and clinical data of patients, such as body mass index or waist circumference, which may provide information on potential obesity. Further analyses will be conducted to investigate these data.

## 5. Conclusions

In conclusion, the FIB4 index assessment is a potential screening tool in the primary care setting. This non-invasive marker is affordable and could identify individuals who need assessment of liver fibrosis, representing a “red flag” for primary care physicians in order to increase awareness of liver diseases. This strategy, combined with further liver stiffness measurements, could improve the diagnosis of early liver disease.

## Figures and Tables

**Table 1:** Biochemical parameters distributed by FIB4 cut-offs (A) in all the population affected (*N* = 31,753) and (B) in the subset of patients aged 40 to 75 years (*N* = 18,102).

(A) FIB4 All the Population		FIB4 < 1.3	FIB4 1.3–3.25	FIB4 > 3.25	*p*
*N* = 31,753	*N*	18,957	11,808	988	
AST (IU/L)	Mean	24.0	26.5	49.4	<0.0001 ^†,‡,§^
	Median	22	23	29	
	Range	4–399	9–828	11–559	
	SD	10.8	19.6	76.0	
ALT (IU/L)	Mean	24.5	22.8	34.5	<0.0001 ^†,‡,§^
	Median	19	18	18	
	Range	3–713	3–740	3–537	
	SD	20.4	32.0	73.8	
Glucose (mg/dL)	Mean	93.8	100.1	99.1	<0.0001 ^†,‡^ ns ^§^
	Median	87	92	90	
	Range	28–500	36–145	49–310	
	SD	29.0	28.2	30.8	
Triglycerides (mg/dL)	Mean	130	123	130.4	<0.0001 ^†^ 0.04 ^‡^ ns ^§^
	Median	105	105	98	
	Range	24–999	24–998	39–984	
	SD	103.5	75.8	89.3	
(B) FIB4 Age 40–75		FIB4 < 1.3	FIB4 1.3–3.25	FIB4 > 3.25	*p*
*N* = 18,102	*N*	11,091	6748	263	
AST (IU/L)	Mean	23.1	28.5	75.3	<0.0001 ^†,‡,§^
	Median	22	25	50	
	Range	6–128	10–307	13–392	
	SD	7.8	18.7	72.3	
ALT (IU/L)	Mean	25.2	26.1	53.0	<0.001 ^†^ <0.0001 ^‡,§^
	Median	21	20	35	
	Range	4–346	3–326	4–398	
	SD	15.7	26.6	63.0	
Glucose (mg/dL)	Mean	99.1	99.0	106.0	ns ^†^ <0.01 ^‡,§^
	Median	90	92	93	
	Range	33–500	38–344	58–310	
	SD	31.5	27.7	41.4	
Triglycerides (mg/dL)	Mean	141.9	125.9	158.7	<0.0001 ^†^ ns ^§^ <0.01 ^§^
	Median	116	105	108	
	Range	26–996	24–998	41–984	
	SD	104.4	84.0	140.7	

^†^*p* FIB4 < 1.3 vs. 1.3 ≤ FIB4 < 3.25; ^‡^ *p* FIB4 < 1.3 vs. FIB4 ≥ 3.25; ^§^ *p* 1.3 ≤ FIB4 <3.25 vs. FIB4 ≥ 3.25. ALT, alanine aminotransferase; AST, aspartate aminotransferase; FIB4, fibrosis 4 score; ns, not significant.

**Table 2:** Summary of data of FIB4 requested and added according to the clinical setting.

Clinical Setting	Total FIB4	FIB4 ≥ 3.25	FIB4 Requested	% FIB4 Requested	FIB4 ≥3.25 Requested	% FIB4 ≥3.25 Requested	FIB4 Ad Hoc	% FIB4 Ad Hoc	FIB4 ≥ 3.25 Ad Hoc	% FIB 4 ≥ 3.25 Ad Hoc
Diabetes	4929	99	658	13.3	21	3.2	4271	86.7	78	1.8
Liver	5077	98	2094	41.2	54	2.3	2983	58.8	44	1.5
Hypercholesterolemia	11,981	169	1203	10.1	35	2.9	10,775	89.9	134	1.2
Arterial hypertension	5392	97	776	14.4	15	1.9	4616	85.6	82	1.8
Thyroid	6079	75	902	14.8	14	1.6	5177	85.2	61	1.2
Basic Health Study	9310	144	1271	13.7	35	2.8	8039	86.3	109	1.4
Anemia	745	13	174	23.4	2	1.1	571	76.6	11	1.9
Total	43,513	695	7081	16.3	176	2.5	36432	83.7	519	1.4

FIB4, fibrosis 4 score.

**Table 3:** Evidence of liver fibrosis in medical records of cases with FIB4 ≥ 3.25 according to FIB4 (requested or not) and its etiology. A: With evidence of fibrosis in the medical record (based on transient elastography, magnetic resonance, and/or sonography results). B: Without evidence of fibrosis in the medical record. C: No medical records available.

	*N*	FIB4 Requested	FIB4 Ad Hoc
Total number of cases with FIB4 ≥ 3.25	263	46 (17%)	217 (3%)
(A) Evidence of fibrosis	52	13 (28%)	39 (18%)
Etiology			
Alcohol	27	7	20 ^†^
HCV	8	0	8
HBV	1	0	1
MAFLD	3	0	3
At least two of T2DM/AH/DL/obesity	10	4	6
Hypercholesterolemia	1	0	1
Other diagnosis	2	2	0
(B) No evidence of fibrosis	190	31 (67%)	159 (73%)
Etiology			
Alcohol	40	5	35 ^‡^
HCV	15	5	10
HBV	4	1	3
MAFLD	13	4	9
At least two of T2DM/AH/DL/obesity	67	10	57
Autoimmune liver disease	2	0	2
Ascites	1	0	1
Arterial hypertension	12	1	11
Hypercholesterolemia	3	1	2
Hypothyroidism	1	0	1
Unclassified thrombocytopenia	2	0	2
Oncologic treatment/hematologic illness	6	1	5
Other diagnosis	24	3	21
(C) Medical records not available	21	2	19

^†^ Two cases with HCV infection and one case with HBV infection; ^‡^ Two cases with HBV infection. Abbreviations: AH, arterial hypertension; DL, dyslipidemia; T2DM, type 2 diabetes mellitus; FIB4, fibrosis 4; HBV, hepatitis B virus; HCV, hepatitis C virus; MAFLD, metabolic-associated fatty liver disease.

## Data Availability

The data that support the findings of this article are available from the corresponding authors on reasonable request.

## Supplementary Materials

The following are available online at https://www.mdpi.com/article/10.3390/diagnostics11122236/s1: Table S1: Primary Care Protocols (PCPs) distribution of FIB4 studies performed, Table S2: Primary Care Protocol (PCP) combinations requested in more than 100 patients.
