# Supplementary material for: Assessing Liver Fibrosis Using the FIB4 Index in the Community Setting
Source: Diagnostics (Basel). 2021 Nov 29;11(12):2236. doi: 10.3390/diagnostics11122236 (PMC8700445; doi:10.3390/diagnostics11122236)
Supplement: Supplementary file 1 [file diagnostics-11-02236-s001.zip › diagnostics-1480537-supplementary.pdf]

diagnostics

MDPI

Supplementary Materials

Table S1. Primary Care Protocols (PCPs) distribution of FIB4 studies performed.

PCPs	Total FIB4	FIB4 <1.3	%FIB4 <1.3	FIB4 1.3-3.25	%FIB4 1.3-3.25	FIB4 ≥3.25	%FIB4 ≥3.25	FIB4 requested	%FIB4 requested	FIB4 ≥3.25 requested	%FIB4 ≥3.25 requested	FIB4 added	%FIB4 added	FIB4 ≥3.25 added	%FIB4 ≥3.25 added
PCP01-Arterial hypertension	5,392	3,093	57.4	2,124	39.4	97	1.8	776	14.4	15	1.9	4,616	85.6	82	1.8
PCP02-Hypercholesterolemia: primary prevention	138	87	63.0	46	33.3	4	2.9	42	30.4	2	4.8	96	69.6	2	2.1
PCP03-Hypercholesterolemia: follow-up/prevention	11,843	7,206	60.8	4,308	36.4	165	1.4	1,164	9.8	33	2.8	10,679	90.2	132	1.2
PCP04- Diabetes mellitus: initial test/follow-up	1,198	691	57.7	453	37.8	27	2.3	182	15.2	5	2.7	1016	84.8	22	2.2
PCP05-Diabetes mellitus:	1,551	911	58.7	581	37.5	28	1.8	156	10.1	6	3.8	1,395	89.9	22	1.6

diagnostics

**annual follow-up**														
**PCP06-Diabetic nephropathy screening**	1,356	793	58.5	507	37.4	31	2.3	195	14.4	8	4.1	1,161	85.6	23	2.0
**PCP07-Hormonal contraception**	6	6	100.0	0	0.0	0	0.0	1	16.7	0	0.0	5	83.3	0	0.0
**PCP08-First trimester pregnancy (33-36 weeks)**	1	1	100.0	0	0.0	0	0.0	0	0.0	0	0.0	1	100.0	0	0.0
**PCP10- Third trimester pregnancy (<13 weeks)**	1	1	100.0	0	0.0	0	0.0	0	0.0	0	0.0	1	100.0	0	0.0
**PCP11-Initial study of anemia**	464	286	61.6	164	35.3	10	2.2	134	28.9	2	1.5	330	71.1	8	2.4
**PCP12-Iron deficiency anemia follow-up (4 months)**	83	61	73.5	20	24.1	1	1.2	20	24.1	0	0.0	63	75.9	1	1.6
**PCP13-First iron deficiency anemia follow-up (4-6 weeks)**	62	47	75.8	12	19.4	2	3.2	14	22.6	0	0.0	48	77.4	2	4.2
**PCP14-Artritis**	1,012	670	66.2	316	31.2	13	1.3	235	23.2	4	1.7	777	76.8	9	1.2
**PCP15-Presurgery**	229	133	58.1	84	36.7	8	3.5	42	18.3	2	4.8	187	81.7	6	3.2

diagnostics

MDPI

**PCP16-Hepatic basic study**	3,192	1,990	62.3	1,107	34.7	51	1.3	239	7.5	7	2.9	2,953	92.5	44	1.5
**PCP17-Acute viral hepatitis**	28	22	78.6	5	17.9	1	3.6	20	71.4	1	5.0	8	28.6	0	0.0
**PCP18-Acute hepatitis follow-up**	515	313	60.8	181	35.1	13	2.5	515	100.0	13	2.5	0	0.0	0	0.0
**PCP19-Chronic liver disease: initial study**	1,255	776	61.8	418	33.3	30	2.4	1,255	100.0	30	2.4	0	0.0	0	0.0
**PCP20-Tyroid function: initial study**	4,398	2,878	65.4	1,421	32.3	58	1.3	618	14.1	9	1.5	3,780	85.9	46	1.3
**PCP21-Hypotyroidism: follow-up**	991	581	58.6	388	39.2	7	0.7	125	12.6	2	1.6	866	87.4	5	0.6
**PCP22-Secondary amenorrhea**	70	65	92.9	4	5.7	0	0.0	13	18.6	0	0.0	57	81.4	0	0.0
**PCP23-Suspected menopause**	73	62	84.9	11	15.1	0	0.0	10	13.7	0	0.0	63	86.3	0	0.0
**PCP24-Basic health study**	9,310	5,824	62.6	3,204	34.4	144	1.5	1,271	13.7	0	0.0	8,039	86.3	109	1.4
**PCP25-Urinary infection diagnosis**	1,070	646	60.4	390	36.4	16	1.5	156	14.6	2	1.3	914	85.4	14	1.5
**PCP26-Urinary infection post-treatment test**	168	104	61.9	60	35.7	0	0.0	23	13.7	0	0.0	145	86.3	0	0.0

**PCP27-Asymptomatic bacteriuria in pregnant**	1	0	0.0	1	100.0	0	0.0	1	100.0	0	0.0	0	0.0	0	0.0
**PCP29-Probed patient urine**	1	0	0.0	1	100.0	0	0.0	0	0.0	0	0.0	1	100.0	0	0.0
**PCP30-B12 deficiency anemia: follow-up (6 months)**	61	39	63.9	16	26.2	0	0.0	2	3.3	0	0.0	59	96.7	0	0.0
**PCP31-Folates deficiency anemia: follow-up (6 months)**	57	36	63.2	17	29.9	0	0.0	1	1.8	0	0.0	56	98.2	0	0.0
**PCP32-Diabetes mellitus: screening**	225	144	64.0	77	34.2	2	0.9	39	17.3	0	0.0	186	82.7	2	1.1
**PCP33- Diabetes mellitus: diagnosis**	599	359	59.9	225	37.6	11	1.8	86	14.4	2	2.3	513	85.6	9	1.8
**PCP34-Subclinic hypothyroidism**	172	100	58.1	68	39.5	3	1.7	67	39.0	1	1.5	105	61.0	2	1.9
**PCP35-Tyroid: start/modification of treatment**	180	109	60.6	63	35.0	3	1.7	32	17.8	0	0.0	148	82.2	3	2.0
**PCP36-Hypertyhyroidism:**	338	218	64.5	111	32.8	4	1.2	60	17.8	2	3.3	278	82.2	2	0.7

diagnostics MDPI

**follow-up**														
**PCP37-Folates deficiency anemia: first follow-up**	8	5	62.5	3	37.5	0	0.0	2	25.0	0	0.0	6	75.0	0	0.0
**PCP38-B12 deficiency anemia: first follow-up**	10	7	70.0	3	30.0	0	0.0	1	10.0	0	0.0	9	90.0	0	0.0
**PCP39-Anemia follow-up in chronic processes**	7	6	85.7	1	14.3	0	0.0	5	71.4	0	0.0	2	28.6	0	0.0
**PCP40-Iron overload confirmation**	87	60	69.0	24	27.6	3	3.4	65	74.7	3	4.6	22	25.3	0	0.0
**Total**	**46,152**	**28,330**	**61.4**	**16,414**	**35.6**	**732**	**1.3**	**7567**	**16.40**	**184**	**2.4**	**38,585**	**83.30**	**548**	**1.4**

In the PCPs related to diabetic pathology (D) (PCP04, 05, 06, 32 and 33) 4929 FIB4 studies were performed. Just 13% of all D studies FIB4 was requested, with a 3.2% FIB4 ≥ 3.25 and 1.8% FIB4 ≥ 3.25 among FIB4 studies added.

In the PCPs related to liver pathology (LP) (PCP16, 17, 18, 19 and 40) 5077 FIB4 studies were performed. A 41% of all LP studies FIB4 was requested, with a 2.6% FIB4 ≥ 3.25 and 1.8% FIB4 ≥ 3.25 among FIB4 studies added.

In the PCPs related to hydoi: 10.1016/j.jhep.2021.05.025percholesterolemia (HC) (PCP02 and 03) 11,981 FIB4 studies were performed. Just a 10% of all HC studies FIB4 was requested, with 2.9% a FIB4 ≥ 3.25 and 1.2% FIB4 ≥ 3.25 among FIB4 studies added.

In the PCP01, related to arterial hypertension (AH) 5392 FIB4 studies were performed. Just a 14% of all AH studies FIB4 was requested, with a 1.9% with FIB4 ≥ 3.25 and 1.8% FIB4 ≥ 3.25 among FIB4 studies added.

In the PCPs related to Thyroid pathology (TP) (PCP21, 34, 35 and 36) 6079 FIB4 studies were performed. Just a 15% of all TP studies FIB4 was requested, with a 1.6% with FIB4 ≥ 3.25 and 1.2% FIB4 ≥ 3.25 among FIB4 studies added.

In the PCPs related to Anemia studies (AS) (PCP11, 12, 13, 30, 31, 37, 38 and 39) 745 FIB4 studies were performed. In the 23% of all AS studies FIB4 was requested, in which 1.1% FIB4 ≥ 3.25 and 1.9% FIB4 ≥ 3.25 among FIB4 studies added.

Finally, in the PCP24, which is a Basic Study of Health (BH), frequently requested as starting study in analytical control of patients, 9310 FIB4 studies were performed. Just a 14% of all BH studies FIB4 was requested with a 2.8% FIB4 ≥ 3.25 and 1.4% FIB4 ≥ 3.25 among FIB4 studies added.

diagnostics

Table S2. Primary assistance protocols (PCPs) combinations requested in more than 100 patients.

**PCPs**	**n**
3	1798
3; 24	968
24	756
1; 3; 24	549
3; 20	438
3; 20; 24	426
1; 3	425
3; 16; 24	267
1; 3; 20; 24	266
3; 16; 20; 24	211
3; 25	195
1; 3; 16; 24	194
1; 3; 16; 20; 24	190
20; 24	139
19	134
3; 21	130

16; 24	116
3; 21; 24	109
20	107
25	101

Among all the 1,871 PCPs combinations, just 20 of them were observed in more than 100 patients, accounting for a total of 7519 patients. Taking this representative subset of patients, in 6166 (82%) of them PCP03 (hypercholesterolemia) was requested. In 3180 of these studies PCP03 was requested together with PCP24 (Basic Health). In 2591 patients PCP01 (Arterial Hypertension) was requested, most of them (2341) joined to PCP03. In fact, in 13,508 (74.6%) of the entire 18,102 patient's cohort (40 to75 years), PCP01 and/or PCP03 were requested, both clinical situations included in MetSd criteria.
